# Job satisfaction of Japanese midwives in the Tokyo metropolitan area

**DOI:** 10.18332/ejm/167388

**Published:** 2023-07-19

**Authors:** Yuko Koga, Noboru Iwata

**Affiliations:** 1 Midwifery Program, Kiryu University, Midori, Japan; 2 Graduate School of Nursing, Dokkyo Medical University, Mibu, Japan

**Keywords:** job satisfaction, midwives, factor analysis, work facility

## Abstract

**INTRODUCTION:**

We explore job satisfaction among Japanese midwives working in different institutions within the Tokyo metropolitan area and relate this to midwives’ age.

**METHODS:**

The study involved a questionnaire survey of 423 midwives working in 113 general hospitals, 70 clinics, and 58 midwifery centers in the Tokyo Metropolitan Area of Japan. The questionnaire consisted of items related to demographic and job satisfaction. Questionnaires were returned by 199 participants (47%).

**RESULTS:**

Of the 199 midwives, 142 worked in general hospitals (71%), 26 worked in maternity clinics (13%), and 31 worked in midwifery centers (16%). Factor analysis extracted six job satisfaction factors labelled as: F1 ‘midwifery services’, F2 ‘interpersonal relations’, F3 ‘rewarding and autonomy’, F4 ‘working environment’, F5 ‘working conditions’, and F6 ‘collaboration with doctors’. Job satisfaction scores for all factors were the highest among midwives working in midwifery centers, followed by those working in maternity clinics and hospitals. Job satisfaction of F1 and F3 of those working in midwifery centers was significantly higher than those working in hospitals and maternity clinics for the younger age group, while the scores of those working in maternity clinics became higher and closer to those working in midwifery centers for other age groups.

**CONCLUSIONS:**

Job satisfaction of midwives varies by different institutions, particularly lowest for those working in hospitals than those working in midwifery clinics and centers. It is necessary to devise useful strategies for midwives in hospitals to enhance their satisfaction with midwifery services and to feel rewarded by and autonomy at work.

## INTRODUCTION

A midwife is a socially responsible professional who provides the necessary support and care for women during pregnancy, childbirth, postpartum period, and newborns^1^. Consensus on midwives is recognized internationally, but the scope of their practice varies considerably across countries^2^. For example, midwives can prescribe medications and handle risky pregnancies and deliveries in Australia, the Netherlands, and the United Kingdom, but not in Japan and Germany^2^. The types of midwifery facilities also vary across countries, similar to permitted treatment and care^3^.

In Japan, the midwifery certification system and work facilities seem unique: 1) all midwives must be licensed nurses; and 2) there are three types of facilities where midwives practice, namely hospitals, maternity clinics, and midwifery centers. According to the Japan Medical Service Law, hospitals have ≥20 beds for inpatients, clinics have ≤19 beds, both are staffed by doctors, and midwifery centers are defined as facilities where normal deliveries are supported by midwives alone. Japanese midwives work mainly in hospitals (60%), followed by maternity clinics (20%) and midwifery centers (6%)^4^.

There has been an increase in mixed wards combining obstetrics with other medical departments to prevent the decrease in bed occupancy resulting from the remarkably declining birth rate in Japan^5^. This means that many midwives in Japan cannot focus solely on midwifery-related work^6^. Thus, early turnover in the young nursing profession has become a problem securing hospital staff in Japan^7^.

Maternity care has become more medicalized, and in some settings, the role of midwives in childbirth has diminished^8,9^. In high-income countries, increased work-related pressures and a lack of opportunities to provide woman-centered care have become more common^10^. These factors can affect job satisfaction, which has been considered a major factor that affects job turnover, absenteeism, and quality of care in health settings^11,12^. A large body of research focuses on job satisfaction in nursing, but limited evidence is related specifically to midwives’ views, experiences, and role satisfaction^9^.

Recent empirical studies have revealed that midwives’ job satisfaction is high in Australia^9^ and the Netherlands, where many midwives are in private practice^13^. A multinational cross-sectional survey in some European and Asian countries reported that job satisfaction was the lowest among midwives within five years of service^12^. In Japan, although it has been reported that midwives working in hospitals have high job satisfaction when the number of deliveries is high^14^, the situation for midwives working in maternity clinics and midwifery centers has not yet been investigated.

Therefore, we conducted a survey on job satisfaction among midwives in the Tokyo metropolitan area and examined and reported the dimensionality of job satisfaction in midwives, the effects of age and other demographics on job satisfaction, and the effect of the types of working facilities.

## METHODS

### Survey and questionnaire

We randomly selected 241 facilities supporting childbirth in Tokyo and Kanagawa Prefecture, Japan, and sent letters explaining the survey’s purpose and a document requesting their cooperation. In addition, we received consent responses from 47 facilities (26 hospitals, five maternity clinics, and 16 midwifery centers) in which 423 midwives worked. We then sent the requesting letters and survey questionnaires to these facilities with return envelopes to the researcher. A total of 423 questionnaires were sent, and 202 responses were returned by mail. After excluding three incomplete responses, 199 were analyzed (47% response rate). We focused on the responses of younger to middle-aged midwives aged <40 years since these age groups have been considered at high risk of stress and burnout, even in the UK^15^.

The survey questionnaire consisted of 23 items on demographics and professional experiences and 51 items on job satisfaction, which were constructed according to the Stamp et al.^16^ Index of Work Satisfaction in Japanese (WSI-J)^17^. It has been confirmed for reliability and validity^17^ and has been used in many studies on Japanese nurses^18-20^. Responses were rated on a five-point Likert scale ranging from 1=‘strongly disagree’ to 5=‘strongly agree’, with higher scores representing higher satisfaction. We modified some of the WSI-J items to be more suitable for midwifery work and added some items. Therefore, we conducted a factor analysis to construct the subscales.

### Statistical analyses

A descriptive analysis was used to describe the characteristics of the study population. Responses to the job satisfaction scale items were analyzed using a principal axis factor analysis. To evaluate the suitability of the data for factor analysis, the Kaiser-Mayer-Olkin (KMO) measure of sampling adequacy (>0.70)^21^ and the Bartlett test of sphericity (p<0.05)^22^ were computed. The following set of rules helped to determine the optimal number of components to be retained^23^: Kaiser’s criterion for components with eigenvalues >1.0, the ratio of the eigenvalue of the first and second unrotated component ≥4.0, Cattell Scree Test, and individual item loadings ≥0.40^24^. Subsequently, a promax rotation was used to clarify the concept of the extracted factors. Cronbach’s α^25^ and McDonald’s ω^26^ were calculated to determine the reliability of each scale based on the factor structure.

As the scale score of each factor, the mean rating score on the items of each factor was calculated and used in the following analyses to account for the difference in the number of items. A general linear model analysis was used to examine the effects of the types of work facilities (hospital, maternity clinic, and midwifery center) and age categories (22–29, 30–34, and 35–39 years) on each scale score while controlling for demographic confounders, such as marital status and having a child. Bonferroni correction was employed for post hoc testing when significant main or interaction effects were found. SPSS version 25 was used for all the analyses.

### Ethical considerations

The researcher explained in writing that cooperation in the survey was voluntary, that no disadvantages would be incurred by declining to participate or not participating in the study, that the questionnaire would be anonymous, it would not identify individuals, and that there would be no financial burden on the subjects. The collection of questionnaires was considered as informed consent for the study. This study was approved by the Ethics Committee of the International University of Health and Welfare, Japan.

## RESULTS

### Demographics of participants

More than two-thirds of the respondents were working in hospitals (n=142; 71%), followed by midwifery centers (n=31; 16%) and maternity clinics (n=26; 13%). The average age of the respondents was 31.9 ± 4.4 years. There were no midwives aged <25 years in maternity clinics and midwifery centers, resulting in significant differences in age distribution according to the type of work facility. In addition, marital status and having children varied significantly; midwives in maternity clinics and midwifery centers were more likely to be married and have children than those in hospitals (p<0.05). The average years of experience as a midwife were 6.9 ± 4.1 (0–18) years. Still, nearly half of those in midwifery centers had more than ten years of experience and thus assisted in more than 200 deliveries (Table 1).

**Table 1:** Demographics of the survey participants

*Characteristics*	*Hospital (N=142)*		*Maternity clinic (N=26)*		*Midwifery center (N=31)*		*Total (N=199)*		*χ^2^*	*p*
	*n*	*%*	*n*	*%*	*n*	*%*	*n*	*%*		
**Age** (years)									10.5	0.032
22–29	52	37	6	23	5	16	63	32		
30–34	49	35	12	46	9	29	70	35		
35–39	41	29	8	31	17	55	66	33		
**Marital status**									7.4	0.025
Married	57	40	16	62	19	61	92	46		
Not Married	85	60	10	39	12	39	107	54		
**Child**									6.3	0.042
Have	41	29	13	50	14	45	68	34		
No child	101	71	13	50	17	55	131	66		
**Years as midwife**									7.1	0.029
<10	109	77	21	81	17	55	147	74		
10–18	33	23	5	19	14	45	52	26		
**Number of assistances with delivery**									15.5	0.001
<100	53	37	5	19	2	7	60	30		
100–199	36	25	7	27	7	23	50	25		
≥200	53	37	14	54	21	70	88	44		

Cross-sectional questionnaire survey, Tokyo metropolitan area, worksite and postal survey, N=199.

### Dimensionality of the job satisfaction scales

The KMO measure of sampling adequacy yielded a coefficient of 0.905, and the Bartlett test of sphericity produced a value of 6672.9 (df=1275, p<0.001), indicating that the sampling adequacy was excellent and the correlation matrix was suitable for factor analysis. In the initial unrotated principal axis analysis, the sum of variances of factor loadings and the explained percentages of these components were: 19.57 (38.4%), 3.58 (7.0%), 2.62 (5.1%), 1.83 (3.6%), 1.50 (2.9%), 1.24 (2.4%), 0.90 (1.8%), 0.76 (1.5%), and so on. Then, six factors were promax rotated, and finally, 45 items were adopted by deleting six items that did not meet the criteria with a factor loading criterion of ≥0.4 (Table 2).

**Table 2:** Promax rotated factor structure of job satisfaction of Japanese midwives (N=199)

	*F1*	*F2*	*F3*	*F4*	*F5*	*F6*
	*Midwifery services*	*Interpersonal relations*	*Rewarding and autonomy*	*Working environment*	*Working conditions*	*Collaboration with physicians*
Cronbach’s alpha	0.957	0.913	0.944	0.812	0.830	0.850
McDonald’s omega	0.937	0.912	0.945	0.811	0.824	0.856
Able to assist with births in a home-like atmosphere	**0.89**	0.09	-0.18	-0.27	-0.02	0.10
Can spend more time on prenatal checkups	**0.89**	0.03	-0.11	-0.03	-0.03	0.09
Assist in near-natural childbirth	**0.85**	-0.09	-0.08	-0.26	0.14	0.16
Normal birth can be performed only by a midwife	**0.85**	0.11	-0.07	-0.36	-0.03	0.05
Can be involved after discharge from the hospital	**0.84**	-0.01	0.08	0.09	-0.25	-0.03
Able to assist with freestyle deliveries	**0.83**	0.00	-0.19	-0.13	0.04	0.26
Adequate breastfeeding and breast care	**0.78**	-0.02	0.01	0.28	-0.03	-0.22
Involvement of the family from the time of conception	**0.77**	0.10	-0.15	0.03	-0.06	0.12
Can spend more time on health guidance	**0.71**	0.00	0.14	0.25	-0.06	-0.18
Take the time to get involved in the birthing process	**0.71**	-0.01	0.07	0.13	0.15	-0.05
Allow more time for postpartum care	**0.70**	-0.01	0.07	0.20	0.11	-0.20
Continuous involvement with pregnancy and postpartum	**0.67**	0.06	0.01	0.01	-0.06	0.03
Feel connected to the community	**0.67**	-0.01	0.11	0.00	-0.06	0.09
I can give you the full care you want to give yourself	**0.64**	-0.02	0.25	0.12	0.04	-0.05
Ample time for staff to talk to each other	**0.48**	0.22	0.09	0.17	0.08	0.01
Greatly demonstrates birthing skills	**0.47**	0.04	0.33	-0.25	0.02	0.07
Have a respected supervisor or colleague	0.11	**0.85**	-0.03	0.06	-0.10	-0.10
Helping and cooperating with each other	0.14	**0.83**	-0.08	0.14	-0.09	-0.08
I feel supported by the people around me	-0.04	**0.82**	0.22	0.05	-0.20	-0.18
Atmosphere of consultation	0.00	**0.66**	0.06	0.03	0.28	-0.16
Atmosphere of easy integration	-0.04	**0.63**	-0.05	-0.08	**0.41**	-0.17
Staff is cohesive	0.19	**0.61**	-0.17	0.32	-0.06	0.08
Atmosphere in which it is easy to express opinions	0.08	**0.55**	0.04	0.01	0.31	-0.07
We can continue to take pride in our work	-0.05	0.08	**0.96**	-0.04	-0.08	0.05
I am glad I became a midwife	-0.05	-0.07	**0.92**	-0.19	0.07	-0.03
Finding the value of existence	0.02	0.15	**0.84**	-0.01	-0.11	0.04
Always feel a sense of fulfillment	0.11	-0.02	**0.78**	-0.07	0.10	-0.01
Always aim for care that satisfies the birthing mother	0.22	-0.06	**0.69**	0.02	0.08	0.04
Continuous improvement	0.16	0.21	**0.42**	0.07	-0.03	0.25
Adequate staffing for emergencies	-0.05	0.20	-0.18	**0.72**	0.03	0.15
Good educational system	-0.16	0.22	0.02	**0.67**	-0.06	0.23
There are enough midwives	0.14	-0.09	-0.06	**0.65**	0.24	-0.01
There are enough doctors	-0.16	-0.05	-0.09	**0.64**	0.10	0.13
Strong collaboration with partner hospitals	0.13	-0.13	0.03	**0.58**	0.10	0.30
Nursing managers consult with staff	-0.08	0.34	0.11	**0.40**	0.07	0.15
Easy access to child care	-0.14	0.03	0.01	0.11	**0.70**	0.20
Easy to take desired holidays	-0.15	0.06	-0.04	0.18	**0.70**	-0.03
Less paperwork	0.27	-0.28	0.10	0.07	**0.69**	-0.06
Satisfied with salary	0.04	0.04	-0.08	0.28	**0.56**	-0.18
Relatively little overtime work	-0.05	-0.09	0.10	0.17	**0.56**	-0.30
Supervisor’s understanding of child care	-0.02	0.30	0.03	-0.10	**0.50**	0.17
Colleagues’ understanding of child care	-0.01	**0.41**	-0.04	-0.09	**0.44**	0.06
Sufficient teamwork	0.13	-0.12	-0.01	0.40	-0.13	**0.88**
Physicians understand and appreciate midwives	0.07	-0.17	0.05	0.29	-0.05	**0.80**
The doctor (supervisor) trusts you	0.19	-0.16	0.25	0.02	0.03	**0.58**
**Inter-factor correlations**						
F1. Midwifery services	1.00	0.57	0.70	0.29	0.48	0.37
F2. Interpersonal relations		1.00	0.60	0.23	0.60	0.44
F3. Rewarding and autonomy			1.00	0.34	0.54	0.37
F4. Working environment				1.00	0.13	-0.22
F5. Working conditions					1.00	0.32
F6. Collaboration with physicians						1.00

Factor 1, ‘Satisfaction with Midwifery Services’, consisted of 16 items, such as a home-like atmosphere for childbirth, ‘naturalistic birth care’, and so on. Factor 2, ‘Satisfaction with Interpersonal Relations’, consisted of seven items, including ‘I help and cooperate’, and ‘I have a supervisor or colleague whom I respect’, and so on. Factor 3, ‘Satisfaction with Rewarding and Autonomy’, consisted of five items, such as ‘I am happy to be a midwife’, ‘I can continue to be proud of being a midwife’, and so on. Factor 4, ‘Satisfaction with Working Environment’, consisted of six items, including ‘there are enough midwives’ and ‘there are personnel and medical equipment that can respond quickly to emergencies’. Factor 5, ‘Satisfaction with Working Conditions’, consisted of seven items, including ‘Easy to take desired holidays’ and ‘Good environment for raising children’. Factor 6, ‘Satisfaction with Collaboration with Doctors’, consisting of three items, such as ‘doctors and midwives work well as a team’ and ‘doctors understand and appreciate midwives’. Cronbach’s α and McDonald’s ω for each factor exceeded 0.81.

The inter-factor correlations indicated that Factor 1, ‘Satisfaction with Midwifery Services’, Factor 2, ‘Satisfaction with Interpersonal Relations’, Factor 3, ‘Satisfaction with Rewarding and Autonomy’, and Factor 5, ‘Satisfaction with Working Conditions’, were highly correlated with each other. However, other factor pairs were also correlated. The inter-factor correlations indicated that Factor 4, ‘Satisfaction with Working Environment’, was relatively independent of Factor 5, ‘Satisfaction with Working Conditions’, and rather in an opposite direction to that of Factor 6, ‘Satisfaction with Collaboration with Doctors’.

### Differences in job satisfaction according to the type of work facility

As shown in Table 3, all six job satisfaction scores varied significantly across the type of work facility, with the highest scores for midwives at midwifery centers, followed by those at maternity clinics and hospitals. On Factor 1, ‘Satisfaction with Midwifery Services’, in addition to the main effect of the type of work facility [F(2,190)=90.19, p<0.001, partial η^2^=0.490], the main effect of age group [F(2,190)=5.53, p=0.005, partial η^2^=0.056] and the interaction effect of these variables [F(4,190)=3.45, p=0.010, partial η^2^=0.068] were significant. Also, age group × type of work facility interaction was significant on Factor 4, ‘Satisfaction with Working Environment’ [F(4,190)=2.60, p=0.037, partial η^2^=0.053] and marginally significant for Factor 3, ‘Satisfaction with Rewarding Autonomy’ [F(4,190)=2.04, p=0.091, partial η^2^=0.042].

**Table 3:** Mean scores on six subscales of job satisfaction according to the type of work facility and age group

*Factors/Work facility^a^*	*Age (years)^b^*									*Effect size*
	*22–29*		*30–34*		*35–39*		*GLM*			
	*Mean*	*SD*	*Mean*	*SD*	*Mean*	*SD*		*F*	*p*	*η_p_^2^*
**F1: Midwifery services**										
Hospitals	2.73	0.72	2.62	0.72	2.60	0.80	a	90.19	<0.001	0.49
Maternity clinics	2.96	0.88	4.04	0.74	4.17	0.44	b	5.53	0.005	0.06
Midwifery centers	4.26	0.98	4.76	0.18	4.63	0.43	a × b	3.45	0.010	0.07
**F2: Interpersonal relations**										
Hospitals	3.49	0.88	3.61	0.74	3.43	0.83	a	23.05	<0.001	0.20
Maternity clinics	3.13	0.70	3.80	0.47	3.89	0.30	b	2.26	0.107	0.02
Midwifery centers	4.37	0.53	4.73	0.25	4.70	0.30	a × b	1.26	0.289	0.03
**F3: Rewarding and autonomy**										
Hospitals	3.24	0.92	3.42	0.90	3.24	0.88	a	35.15	<0.001	0.27
Maternity clinics	3.36	0.39	3.89	0.66	4.29	0.35	b	2.04	0.132	0.02
Midwifery centers	4.63	0.45	4.85	0.13	4.57	0.41	a × b	2.04	0.091	0.04
**F4: Working Environment**										
Hospitals	3.20	1.01	3.03	0.93	2.87	0.70	a	3.85	0.023	0.04
Maternity clinics	2.69	0.56	3.32	0.55	3.63	0.41	b	1.78	0.172	0.02
Midwifery centers	3.23	0.72	3.98	0.21	3.33	0.44	a × b	2.60	0.037	0.05
**F5: Working conditions**										
Hospitals	2.83	0.90	3.08	0.70	2.89	0.83	a	18.29	<0.001	0.16
Maternity clinics	3.44	0.70	3.16	0.50	3.17	0.65	b	1.47	0.232	0.02
Midwifery centers	3.43	0.66	4.28	0.43	4.08	0.71	a × b	0.69	0.603	0.01
**F6: Collaboration with physicians**										
Hospitals	2.78	0.97	2.82	0.90	2.69	0.90	a	18.30	<0.001	0.16
Maternity clinics	3.28	0.80	3.56	0.77	3.54	0.89	b	0.84	0.435	0.01
Midwifery centers	3.53	1.12	3.89	0.58	3.89	0.54	a × b	0.29	0.884	0.01

GLM with controlling for demographic confounders (marital status and having child). As a measure of effect size, partial η^2^ refers to the proportion of variance accounted for, where 0.01, 0.06, and 0.14 represent small, medium, and large effects, respectively. SD: standard deviation.

**Figure 1:**
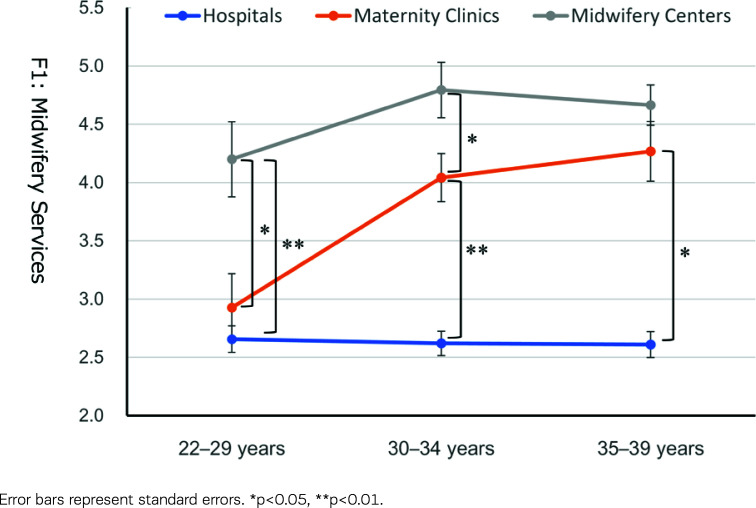
Midwifery Services (Factor 1) scores according to the type of work facility and age group

**Figure 2:**
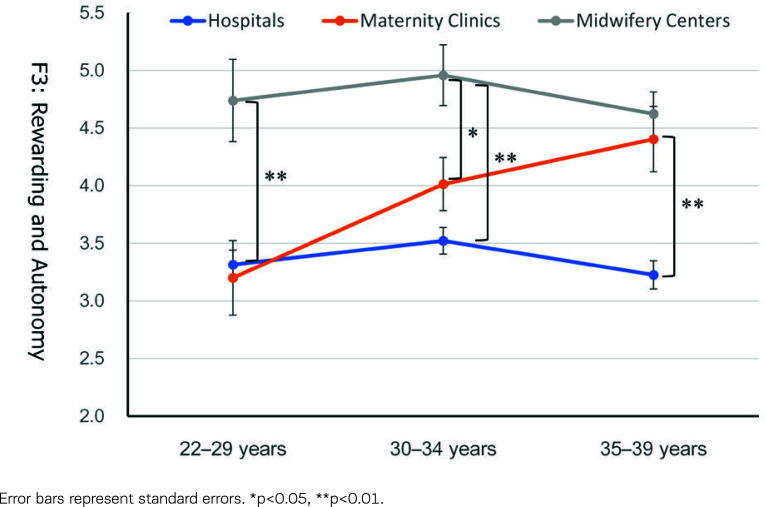
Rewarding and Autonomy (Factor 3) scores according to the type of work facility and age group

The *post hoc* tests for Factor 1, ‘Satisfaction with Midwifery Services’, indicated that the scores of the midwives working in maternity clinics were comparable to those working in hospitals for the younger age group (22–29 years) but were significantly higher than the latter midwives for other age groups (30–34 and 35–39 years) (Figure 1). The scores of those working in midwifery centers were significantly higher than those working in hospitals and maternity clinics for the younger age group. In contrast, the scores of those working in maternity clinics were higher and closer to those working in midwifery centers for other age groups. The corresponding tendency was observed in Factor 3, ‘Satisfaction with Rewarding and Autonomy’ (Figure 2).

**Figure 3:**
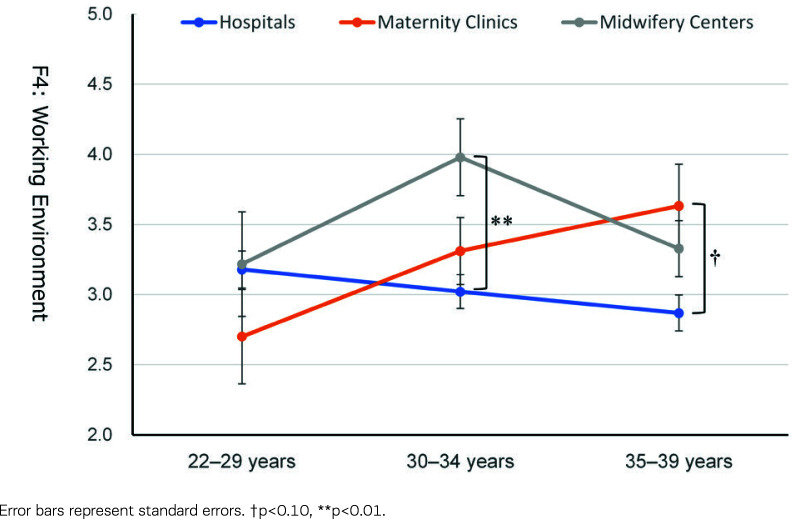
Working Environment (Factor 4) scores according to the type of work facility and age group

The post hoc tests for Factor 4, ‘Satisfaction with Working Environment’ (Figure 3), revealed that scores were comparable among the three types of work facilities for the younger age group. However, the scores of midwives working in hospitals were significantly lower than those working in midwifery centers for the age group of 30–34 years and those working in maternity clinics for the age group of 35–39 years.

## DISCUSSION

This study explored the job satisfaction of Japanese midwives in the Tokyo metropolitan area using a mailed questionnaire survey. Midwives’ job satisfaction varied by type of work facility, with midwifery centers showing the highest job satisfaction, followed by maternity clinics and hospitals (Table 3). A detailed examination revealed that all aspects of job satisfaction differed by type of facility. Still, there were significant or marginal age group × type of work facility interactions on the satisfaction concerning ‘Midwifery Services’, ‘Rewarding and Autonomy’, and ‘Working Environment’. Midwives working in midwifery centers showed consistently high satisfaction, and midwives working in hospitals showed stably lower satisfaction through their 20s and 30s. In contrast, the satisfaction of those working in maternity clinics increased with age (Table 3, Figures 1–3).

One reason for the lower satisfaction of midwives working in hospitals might be the diversification of work due to mixed wards. Approximately 80% of hospitals that handle deliveries in Japan have mixed wards of obstetrics and non-obstetrics departments^27^. Midwives working in such mixed wards are often primarily engaged in nursing rather than midwifery work since all midwives in Japan are licensed nurses, even if they are employed as midwives^28^. The inability to focus on midwifery work and the situation surrounding midwives working in hospitals leads to lower motivation and work satisfaction^29^.

Another reason may be that hospitals are prone to medical intervention, and deliveries tend to be physician-driven^8^. Maternity care has become more medicalized, and in some settings, the role of midwives in childbirth has diminished^30^. However, the inability to fully demonstrate expertise is thought to have led to lower job satisfaction. An obstetric-driven approach to care might lead to a lack of autonomy and lower job satisfaction in midwives^31,32^. It is also difficult for midwives to establish a professional identity in physician-led facilities^33^. Although high job satisfaction has been reported for midwives in Australia^9^ and the Netherlands^13^, most midwives work in private practice.

Midwives working in midwifery centers are the ‘main players’ in childbirth, while those working in hospitals and maternity clinics are ‘assistants’. Therefore, even if their daily work is challenging, midwives in maternity clinics understand their role in childbirth and accept their difficulties. They might be satisfied with their daily practices, establishing their professional identity as midwives. The higher job satisfaction observed in this study could align with reports from other countries that surveyed midwives in birth centers^9,13^.

Clinics in Japan are ‘hospital-like facilities’ with less than 20 beds for inpatients, and maternity clinics specialize in childbirth. Therefore, midwives working in maternity clinics, particularly those with less experience, must become familiar with physician-driven delivery treatment methods similar to those in hospitals. Nevertheless, midwives in maternity clinics have focused chiefly on pregnancy and childbirth. Thus, it seems plausible that midwives working in maternity clinics can gradually feel reward and satisfaction in their daily practice as they have spent a certain amount of time. This may also lead to developing a professional identity as a midwife. Significant changes in satisfaction with ‘Midwifery Services’ (Figure 1) and ‘Rewarding and Autonomy’ (Figure 2) from their 20s to late 30s might reflect these processes.

Since low job satisfaction leads to inadequate professional identity formation and turnover^34^, there is concern about turnover among hospital-employed midwives. To prevent turnover, managers must understand the midwives’ unique expertise and develop organizational strategies to motivate them^35^. In addition, managers should improve their work content and environment to demonstrate their expertise and adjust their workload fully, so they do not become overburdened.

Midwives play a role in measures against the declining birthrate as experts in childbirth and child-rearing^4^. Their ongoing support for expectant and nursing mothers is important for preventing postpartum depression^36^. We believe that further research is needed on creating workplaces where midwives can fully demonstrate their expertise to ensure that they can stay with the organization and ensure the quality of care.

### Limitations

This study has some limitations. First, our sample was limited to midwives aged <40 years, and it was impossible to compare job satisfaction with older age groups or analyze changes in job satisfaction as a result of continued employment. Second, our survey site was limited to the Tokyo metropolitan area, and there may be some bias in the characteristics of working midwives. Third, in the comparison of age groups between hospitals, maternity clinics, and midwifery centers, the sample was considerably smaller for clinics and midwifery centers than for hospitals, and we believe that further evaluation and study with a larger sample are needed in the future to increase the reliability of the results of the analysis. It would be useful to obtain a more detailed picture of the job satisfaction of Japanese midwives and the factors that hinder it through qualitative research.

## CONCLUSIONS

Job satisfaction of midwives varies by different institutions, particularly lowest for those working in hospitals than those working in midwifery clinics and centers. It is necessary to devise useful strategies for midwives in hospitals to enhance their satisfaction with midwifery services and to feel rewarded by and autonomy at work.

## Data Availability

The data supporting this research are available from the authors on reasonable request.
